# Insights into Natural Products in Inflammation

**DOI:** 10.3390/ijms19030644

**Published:** 2018-02-25

**Authors:** Paula B. Andrade, Patrícia Valentão

**Affiliations:** REQUIMTE/LAQV, Laboratório de Farmacognosia, Departamento de Química, Faculdade de Farmácia, Universidade do Porto, Rua de Jorge Viterbo Ferreira n.º 228, Porto 4050-313, Portugal; pandrade@ff.up.pt

The inflammatory process comprises immune, vascular and cellular biochemical reactions, involving different cells, enzymes, cytokines, eicosanoids and transcription factors. It includes the triggering of signaling cascades, the activation of transcription factors, gene expression, increased levels of inflammatory enzymes, and the release of various oxidants and pro-inflammatory molecules in immune or inflammatory cells. At a damaged site, inflammation is initiated by the migration of immune cells from blood vessels and the release of mediators, followed by the recruitment of inflammatory cells and the release of reactive oxygen species (ROS), reactive nitrogen species (RNS) and pro-inflammatory cytokines, to eliminate foreign pathogens, resolve infection and repair injured tissues. As such, inflammation is beneficial for the host, normally being rapid and self-limiting. Deregulated or chronic inflammatory processes, on the other hand, are in the origin of several pathological conditions, such as asthma, cardiovascular and neurodegenerative diseases, among many others. The search for new alternatives to interfere with key players in inflammation has revealed nature to be a prolific source. The special issue “Natural Anti-Inflammatory Agents” includes twenty-seven works offering interesting insights into the involvement of natural extracts and compounds with different targets of this network. The value of this issue is reflected by the diversity of chemical classes and approaches that are addressed. The main findings and advances achieved in these works are pointed out below.

Polyphenols are among the most interesting molecules in nature, and are ascribed several biological activities, including anti-inflammatory properties. Flavonoids, in particular, constitute one of the most represented classes. The neuroprotective effect of licochalcone A, a flavonoid isolated from licorice, was first revealed to be associated with microglia and to have anti-inflammatory effects in Parkinson’s disease models, affecting several targets [1]. It suppressed neuroinflammation by blocking the extracellular signal-regulated kinase (ERK) 1/2 and nuclear factor (NF)-κB p65 pathways in microglia. Licochalcone A suppressed inducible nitric oxide synthase (iNOS) and cyclooxygenase 2 (COX-2) expression and also NO and prostaglandin E2 (PGE2) production in lipopolysaccharide (LPS)-activated BV-2 microglial cells. In addition, it inhibited the production of the pro-inflammatory cytokines tumor necrosis factor α (TNF-α), interleukin (IL)-1β and IL-6.

Tamayose and colleagues evaluated the potential of a flavone derivative (isolated from *Peperomia obtusifolia* (L.) A. Dietr.) as anti-venom therapy for the treatment of snake bite [2]. 8-*C*-Rhamnosyl apigenin neutralized the effect of *Crotalus durissus terrificus* secretory phospholipase A2 (PLA2), by inhibiting the major enzymes involved in arachidonic acid metabolism and by preventing the toxic effects of lipid peroxidation.

The anti-inflammatory properties of epigallocatechin 3-gallate (EGCG), a flavan-3-ol, were used to validate a space- and cost-effective organ culture model that considers the main hallmarks of degenerative intervertebral discs [3]. EGCG inhibited the expression of inflammatory markers (IL-6, and IL-8), collagenases (matrix metalloproteinases (MMP) 1, and MMP3), and pain mediators (iNOS, and prostaglandin-endoperoxide synthase 2 (PTGS2)) in nucleus pulposus (NP) tissue challenged with the diffusion of IL-1β and TNF-α. Besides demonstrating, for the first time, the protective effect of EGCG on inflammatory intervertebral disc tissue, the results provided evidence of the suitability of inflammatory NP cultures for evaluating the effects of potential molecular therapeutics.

*Sambucus nigra* L. is a species with a long use in traditional medicine. Ho and collaborators developed the first study comparing the inhibitory effects of several elderberry and elderflower extracts on NO production in LPS-activated RAW 264.7 macrophages and murine dendritic D2SC/I cells [4]. In addition, they also assessed the effect of some of the phenolic compounds found in them (that included hydroxycinnamic acids, flavonols, flavan-3-ols, anthocyanins, and proanthocyanidins) and of some of their metabolic products. Some considerations regarding composition–activity and structure–activity relationships could be made. Overall, quercetin, rutin, and kaempferol displayed stronger inhibition of NO production, with caffeic acid and 3,4-dihydroxyphenylacetic acid being the most interesting phenolic metabolites.

*Sanghuangporus sanghuang* (Sheng H. Wu, T. Hatt. and Y.C. Dai) is a popular mushroom in oriental countries, and has been traditionally used as food and medicine for thousands of years. Its anti-inflammatory properties were confirmed, for the first time, by testing the effect of a hydroethanol extract obtained from the mycelium on a mouse model of LPS-induced acute lung injury [5]. The extract was revealed to be particularly rich in phenolic acids, reducing the release of pro-inflammatory cytokines and inflammatory substances from cells, up-regulating the expression of antioxidant enzymes, and removing ROS.

Proanthocyanidins (or condensed tannins) are also polyphenols most represented in nature. Zhan and colleagues addressed the anti-inflammatory properties of a grape seed proanthocyanindin extract, focusing on its capacity to prevent ischemia/reperfusion-induced acute renal injury and chronic fibrosis [6]. The protective effects were associated with the blocking of nucleocytoplasmic translocation and the release of high-mobility group box 1 (HMGB1) protein that acts as a damage-associated molecular pattern molecule. Procyanidin A2 (epicatechin-(4β-8, 2β-*O*-7)-epicatechin), an A-type proanthocyanidin mainly found in cranberries and lingonberries, was screened for its ability to manage airway inflammation, which is relevant for allergic asthma [7]. Its capacity to modulate the eosinophil chemokine eotaxin-3 (CCL26) and to interact with the modulator interferon γ (IFNγ) were checked in A549 human lung epithelial cells insulted with IL-4. Procyanidin A2 inhibited IL-4-induced production of CCL26, the greatest efficacy being observed when exposing cells 2–3 h prior to the inflammatory insult. The results did not provided evidence of cooperative inhibition with IFNγ, which displayed a different temporal profile.

There has long been interest in pomegranate, covering issues from nutrition to therapeutics. The bioguided fractionation of a 70% acetone extract of pomegranate peel led to the identification of hydrolysable tannins. Multiple pathogenic mechanisms of acne vulgaris were considered to establish an anti-acne system model, in which both extract and isolated compounds were explored [8]. Therapeutic benefits resulted from the antibacterial activity, namely down-regulation of sebum production by the reduction of lipase activity, attenuation of the inflammatory status by reduction of NO, PGE2, IL-8 and TNF-α production, and avoidance of keratinocyte over-proliferation.

Rhein is a phenolic compound found in the roots of rhubarb (*Rheum palmatum* L. or *Rheum tanguticum* Maxim). Huang and Chan provide the first report on the embryonic toxicity of this anthraquinone, by inducing apoptosis in the inner cell mass of mouse blastocysts, decreasing embryonic development and viability [9]. Rhein-treated blastocysts showed increased levels of ROS and apoptotic signaling involved caspase-9 and caspase-3. In vivo exposure to rhein decreased the mRNA levels of innate immune-related genes (*CXCL1*, *IL-1β* and *IL-8*), but increased the ROS content and transcription levels of antioxidant enzymes (catalase, glutathione peroxidase, Cu/Zn-superoxidase dismutase and Mn-superoxide dismutase).

Resveratrol, a plant polyphenol found in red wine and in various food products, has received great attention in the past years, due to its promising effects in cells and animals. Although this stilbene is already marketed as a dietary supplement, the results obtained in humans are not consistent. A non-targeted metabolomic analysis of the changes caused by treatment with a high dose of resveratrol was performed [10]. Analysis of plasma, urine, and adipose and skeletal muscle tissues of men with metabolic syndrome revealed that the metabolites associated with amino acid and lipid metabolism were particularly affected. The most interesting changes were displayed in the glycolysis, gluconeogenesis, and pyruvate metabolism pathway, pentose phosphate pathway, steroid hormone pathway, and fatty acid pathway. Some of the subtle effects noticed escape routine analyses. In addition, Takeda and collaborators [11] reviewed the peripheral and central mechanisms explaining the effects of resveratrol on nociceptive neuronal activity associated with the relief of nociceptive and inflammatory pain, which are related to its capacity to suppress neuronal excitability, including nociceptive sensory transmission. The contribution to the relief of nociceptive and/or pathological pain in light of recent works was also discussed, pointing out that resveratrol attenuates inflammation-induced mechanical hyperalgesia via the inhibition of peripheral and central COX cascade signaling pathways.

Glucosinolates are sulfur-containing secondary plant compounds characteristic of the Brassicaceae family. Their breakdown products, isothiocyanates, have been implicated in the beneficial properties of those species. Sturm and Wagner provided an overview of the capacity of isothiocyanates to modulate inflammation by inducing the nuclear factor erythroid 2-related factor 2 (Nrf2) and suppressing NF-κB, also exploring the epigenetic mechanisms that may contribute to the health-promoting effects [12]. Sita and colleagues addressed a more specific issue, focusing on the potential role of isothiocyanates in Parkinson’s disease (PD), as a result of in vitro and in vivo studies [13]. The main mechanisms involved in the neuroprotective activity were identified: isothiocyanates are potent Nrf2 activators, with an antioxidant effect via the upregulation of antioxidant response element (ARE)-driven genes, and decreasing the inflammatory response through the NF-κB pathway. Subedi and collaborators used three different systems, comprising microglia, neurons, and astrocytes, to explore for the first time the involvement of allyl isothiocyanate in neuroinflammation and neuroprotection [14]. This compound revealed promising effects, reducing the production of inflammatory mediators and pro-inflammatory cytokines, decreasing NF-κB-mediated transcription, and inhibiting c-Jun N-terminal kinase (JNK) and I-κB phosphorylation following LPS-activated, microglia-induced neuroinflammation.

A cigarette-smoke-exposed mouse model was used to assess the effect of pyranopyran-1,8-dione on lung inflammation [15]. This compound, found in the fruit of *Vitex trifolia* subsp. *litoralis* Steenis, was able to reduce the production of pro-inflammatory cytokines (TNF-α, IL-6) and of keratinocyte chemokine (CXCL1), to diminish the number of immune cells (lymphocytes, macrophages, and neutrophils) infiltrated in lung tissue, and to decrease morphological changes (peribronchial inflammation and airspace enlargement) in lung tissue.

*Ginkgo biloba* L. has long been recognized as a leading medicinal species. The anti-inflammatory properties of ginkgolide A, one of its diterpene trilactones, were first extensively studied, and the underlying molecular mechanism clarified, using LPS-stimulated macrophages in vitro, as well as an in vivo mouse model [16]. It was found that ginkgolide A is able to modulate NF-κB, mitogen-activated protein kinases (MAPKs) (p38 and, ERK), and AMP-activated protein kinase pathways, downregulating NO, COX-2, TNF-α, IL-1β, and IL-6.

Ruscogenin is an important steroid sapogenin found in the roots of *Ophiopogon japonicus* (Thumb.) Ker-Gawl. The effect of this compound on blood–brain barrier (BBB) dysfunction and inflammasome activation is reported here for the first time [17]. Ruscogenin proved to ameliorate ischemia-hypoxia-induced BBB disruption, by upregulating the expression of tight junction proteins and suppressing the expression of IL-1β and caspase-1, modulating the thiredoxin-interactive protein/nucleotide-binding domain (NOD)-like receptor family, pyrin domain containing 3 (TXNIP/NLRP3) inflammasome activation and MAPK pathway.

Nie and colleagues revisited the potential of non-starch polysaccharides (NSPs) to prevent and to help in the therapy of inflammatory bowel disease [18]. Different glucans, fucoidan, mucilage, arabinoxylans, arabinogalactans, prebiotics, gums, pectins and modified NSP, their structures, sources, and mechanisms affecting this condition were discussed.

The use of mixtures instead of isolated compounds may be an advantage when searching for therapeutic alternatives: besides the interactions that may occur between the different constituents, there is an increased possibility of interference with several targets. The immunosuppressive effect of the essential oil of *Litsea cubeba* L. was demonstrated for the first time [19]. Contact hypersensitivity and infiltrative T cells were reduced in a mouse model, as well as the amounts of TNF-α and IL-12 produced by LPS-induced dendritic cells. Citral was implicated in these effects.

A different approach was presented by Almatrafi and collaborators, who evaluated the effects of the ingestion of *Moringa oleifera* Lam. leaves in a guinea pig model of hepatic steatosis [20]. Although no direct relationship with phytochemicals was established, biochemical and histological determinations provided evidence of the interference of this species with key genes involved in lipid synthesis, resulting in lower concentrations of lipids and of inflammatory cytokines in the liver.

The anti-inflammatory activity of extracts of *Cleome rutidosperma* DC. and *Euphorbia thymifolia* L., two species used in traditional medicine, was studied using LPS-stimulated microglial cell line BV2 [21]. The underlying mechanisms were elucidated for the first time and involve the down-regulation of pro-inflammatory gene expression, induction of antioxidant gene expression and modulation of the activation of MAPK and NF-κB. Nevertheless, no phytochemical study was made to support these findings.

Despite the acknowledged value of phytochemicals, their therapeutic efficacy presents some challenges. Issues like solubility, stability, bioavailability, and safety may preclude their clinical application. Conte and colleagues [22] discussed the recent alternatives offered by nano-sized systems for the delivery of polyphenols, phytocannabinoids, phytosterols, carbohydrates, essential oils, and terpenoids, to prevent and/or treat inflammatory diseases, addressing the relationship between the structure and properties of the nanocarrier and release behavior.

In addition to vegetal species, the biomedical potential of marine organisms has been gaining significance in natural products research. Silva and collaborators provided the first study on the anti-inflammatory effects of two edible sea anemones, as well as on their potential cytotoxicity [23]. The results proved the preventive effects of sub-toxic concentrations of the extracts on LPS-induced increased production of NO and ROS by macrophages, and their capacity to inhibit PLA2. The major compound found in the extracts, homarine (an alkaloid), contributed to these effects.

The impact of physiological molecules found in the human body was also explored. The anti-inflammatory properties of irisin, an adipomiokine mediator of physical activity, were revealed to be associated with the downregulation of downstream pathways of toll-like receptor 4 (TLR4)/MyD88, which is connected with the suppressed phosphorylation of MAPK and consequent lower NF-κB activation [24]. Lactoferrin has also attracted attention as an antimicrobial and modulatory agent of inflammatory response. The capacity of this glycoprotein to control inflammatory processes, particularly those associated with infections, was revisited, considering the extensive body of knowledge taken from in vitro and in vivo studies and from the increasing number of clinical trials [25,26]. Hoeke and co-workers provided evidence of the reduction of systemic inflammation by hematopoietic expression of IL-37 under low-grade inflammatory conditions, as indicated by lower circulating immune cells in vivo and lower secretion of pro-inflammatory cytokines by peritoneal macrophages ex vivo, though without effect on hypercholesterolemia and atherosclerosis development [27].

The broad involvement of natural molecules in inflammatory processes and their potential to overcome them, as a consequence of their advantageous, multi-target mechanisms of action, is presented in this special issue. This provides an interesting background for the understanding of how natural molecules act, also contributing to the development of new, naturally-based therapeutic strategies and to the establishment of research models.
